# No specific role for the manual motor system in processing the meanings of words related to the hand

**DOI:** 10.3389/fnhum.2013.00011

**Published:** 2013-02-01

**Authors:** Natasha Postle, Roderick Ashton, Ken McFarland, Greig I. de Zubicaray

**Affiliations:** ^1^Redcliffe-Caboolture Child and Youth Mental Health Service, Queensland HealthCaboolture, QLD, Australia; ^2^School of Psychology, University of QueenslandBrisbane, QLD, Australia

**Keywords:** action representations, embodied language, motor system, word meaning

## Abstract

The present study explored whether semantic and motor systems are functionally interwoven via the use of a dual-task paradigm. According to embodied language accounts that propose an automatic and necessary involvement of the motor system in conceptual processing, concurrent processing of hand-related information should interfere more with hand movements than processing of unrelated body-part (i.e., foot, mouth) information. Across three experiments, 100 right-handed participants performed left- or right-hand tapping movements while repeatedly reading action words related to different body-parts, or different body-part names, in both aloud and silent conditions. Concurrent reading of single words related to specific body-parts, or the same words embedded in sentences differing in syntactic and phonological complexity (to manipulate context-relevant processing), and reading while viewing videos of the actions and body-parts described by the target words (to elicit visuomotor associations) all interfered with right-hand but not left-hand tapping rate. However, this motor interference was not affected differentially by hand-related stimuli. Thus, the results provide no support for proposals that body-part specific resources in cortical motor systems are shared between overt manual movements and meaning-related processing of words related to the hand.

One assumption of late nineteenth century models of language comprehension was that representations of word meaning are distributed throughout the human brain. Both Freud (1891) and Lichtheim (1885) incorporated this assumption in their models that emerged contemporaneously with an emphasis on cortical localization of other functions, such as those involving motor representations (see Henderson, 1992; e.g., Ferrier, 1874). Over a century later, this assumption about the representation of word meaning has been subject to several proposed modifications. One such proposal is that action-related word meanings are necessarily mediated by the somatotopic organization of the motor cortex, and accessed automatically during conceptual processing, i.e., that semantic and motor systems are functionally interwoven (e.g., Gallese and Lakoff, 2005; Pulvermüller, 2005).

A plethora of neuroimaging and electrophysiological studies have shown that motor cortex activity can occur in association with language comprehension. However, the mechanisms responsible for the motor cortex activity observed in these studies remain contentious. A number of authors propose that this activity reflects motor simulation or imagery that is context-dependent or epiphenomenal, reflecting the flow of activation between essentially separate conceptual and motor systems (e.g., Mahon and Caramazza, 2008; Postle et al., 2008; Willems and Casasanto, 2011). In their critique of embodied language theories, Mahon and Caramazza (2008) provide an illustration of a *necessary involvement* of motor systems in representing the meanings of actions: “The process of retrieving the concept HAMMER would itself be constituted by the retrieval of (sensory and motor) information about how to use hammers (i.e., swinging the arm, grasping the object, coordinating the visuo-motor relationships between the nail and the head of the hammer, etc.)” (p. 60). However, as Mahon and Caramazza note, simply observing that the motor system can be activated by action words in a neuroimaging study cannot address this issue.

Despite advances in neuroimaging technologies, or perhaps because of them, behavioral paradigms remain the method of choice for investigating the structural properties of language and organization of semantic memory. This is because it is generally accepted that correlational methods such as neuroimaging are unable to provide unambiguous support for a *necessary involvement* of motor systems in the representation of action word meaning (e.g., Mahon and Caramazza, 2008; Shebani and Pulvermüller, 2013). Although theoretically capable of supporting causal inferences, both lesion-symptom mapping studies and virtual lesioning investigations using cortical stimulation techniques have to date produced equivocal results (e.g., Pulvermüller et al., 2005; Tomasino et al., 2008; Papeo et al., 2009, 2010, 2011; Arévalo et al., 2012; Kemmerer et al., 2012; see Shebani and Pulvermüller, 2013).

One behavioral method employed frequently to establish patterns of motor system involvement in action meaning representation is the go/no-go semantic matching paradigm (e.g., Buccino et al., 2005; Lindemann et al., 2006; Sato et al., 2008; Mirabella et al., 2012). In this paradigm, participants are instructed to make a hand movement to a target on a go-signal, contingent upon the presentation of prime words denoting action meanings; they are to withhold a response (no-go) to non-action related (e.g., abstract) words. A typical finding is that responses with the preferred hand are slower and less accurate for hand-related action words than unrelated action items (e.g., foot-related words; Sato et al., 2008; Mirabella et al., 2012). Similar findings have been reported for names of body-parts (e.g., Lindemann et al., 2006; Experiment 3). These findings have been used to support inferences about the necessary involvement of motor meaning representations and their modularity. However, it is worth noting that the go/no-go paradigm first entails word recognition followed by retrieval of the meaning of the word and its grammatical characteristics prior to the meaning integration required for the matching decision (e.g., Neely, 1991). The matching decision then determines the go response. In addition, the go/no-go investigations have invariably employed a high relatedness proportion for their go condition (i.e., 50% of action words denoted manual movements). High relatedness proportions are known to induce expectancy sets that participants use to strategically enhance their performance in semantic matching tasks (see Neely, 1991). Hence, any influence on go-responses is arguably post-lexical in nature and invoked solely for the purpose of performing the task. Post-lexical motor effects such as these can be explained by spreading activation mechanisms (e.g., Mahon and Caramazza, 2008). Evidence consistent with a post-lexical meaning integration mechanism invoked solely for the purpose of performing the task is provided by Mirabella et al. (2012; Experiment 4), who failed to observe the expected effects of action word category when participants were instead required to respond to the color in which the action words were printed. Similarly, Lindemann et al. (2006; Experiment 4) also failed to observe the expected effects of category with body-part names when the task was letter identification. These differential task effects are not consistent with the hypothesis that body-part specific action meanings are accessed rapidly and automatically by the motor system on word presentation (e.g., Pulvermüller, 2005).

Another behavioral method employed to establish patterns of motor system involvement in action meaning representation is the dual-task paradigm. This interference methodology is based on the premise that when two tasks involving the same cerebral resources are performed concurrently, performance on the tasks is impaired compared to when they are performed alone (Bowers et al., 1978). One of the advantages of the dual task paradigm over the go/no-go paradigm is that no matching task is necessary. Another is that the same response is required for all classes of stimuli (action and non-action). For example, Shebani and Pulvermüller (2013) had 15 participants perform rhythmic movements (paradiddles) of either the hands or the feet paced to a metronome while concurrently performing a working memory task involving recall of concordant arm- and leg-related action word series, compared to no working memory and articulatory suppression conditions (i.e., a 4 × 2 repeated measures design). They reported that hand and foot movements differentially interfered with working memory for words denoting actions performed with those body-parts; a finding that they interpreted in terms of the necessary involvement of motor systems in representing action meaning. By contrast, over five separate experiments, Pecher (2013) found that while concurrent motor (hand grip actions) and verbal (reciting syllables) tasks interfered with visual working memory generally, working memory effects were not more pronounced for pictures of hand manipulable vs. non-manipulable objects.

The dual-task studies by Shebani and Pulvermüller (2013) and Pecher (2013) were primarily concerned with demonstrating motor interference effects on working memory for action-related stimuli. However, if motor systems are necessarily involved in representing action word meanings, then conceptual processing of action words should interfere with motor performance as they share the same neural resources. This is essentially the same logic motivating the abovementioned go/no-go studies. Although Shebani and Pulvermüller (2013) apparently recorded their participants' movement rates, they did not report these results. In order to address this question, Rodriguez et al. (2012; Experiment 2) investigated finger tapping performance while their participants performed concurrent verbal fluency tasks (retrieving words from categories denoting hand manipulable objects vs. “non-motor” objects, e.g., animals). Considerable evidence amassed over several decades of research indicates that right hand motor performance (mediated by left hemisphere motor areas) is significantly reduced while participants perform a concurrent verbal task (due to mediation by left hemisphere language regions; for review see Medland et al., 2002). This robust effect is known as the lateralized dual task decrement, and can serve as a manipulation check by showing that verbal tasks interfere with motor performance to a significant degree. Surprisingly, Rodriguez et al. failed to observe this effect for their hand manipulable objects category, i.e., performance did not differ significantly from the baseline tapping-only condition for either hand, nor did object name generation differ from baseline during tapping (cf. Shebani and Pulvermüller, 2013). However, they did observe a marginally significant reduction in tapping performance for their non-motor category fluency condition that they interpreted in terms of a facilitation effect compared to the manipulable object category.

The present series of three experiments utilized a dual task paradigm to determine whether manual motor systems are necessarily and specifically involved in processing hand-related word meanings. Note that for right-handed individuals, one would expect to find that processing *any* words would result in greater right hand compared to left hand motor performance decrements (consistent with the lateralized dual task decrement; Bowers et al., 1978; Medland et al., 2002), yet the greatest decrement in right hand motor performance should occur for hand-related words compared to other body-part related words if motor systems are differentially and somatotopically involved in the processing of words with body-part specific action meanings (cf. Rodriguez et al., 2012). This is because, in addition to the left hemisphere being involved generally in mediating language functions and right hand motor performance, the hand area of the left motor cortices would be specifically mediating both hand movement and hand-related word processing (e.g., Shebani and Pulvermüller, 2013). By comparison, for other action words there should be a less pronounced decrement in right hand performance, as other areas of the motor cortices should be involved in representing their meanings. Across all experiments, right handed participants performed a finger tapping task that did not involve visual guidance with their left and right hands and was commenced before a verbal task, followed by a post experiment memory test of the presented words (Lomas, 1980; Hellige and Longstreth, 1981; Medland et al., 2002; Boulenger et al., 2006). The concurrent verbal task involved reading body-part names and related action words in infinitive form for the hand (e.g., *hand, grab*), mouth (*mouth, bite*), and foot (e.g., *foot, kick*) in addition to non-human body-part control words (e.g., *tail, wag*). Names of body-parts were included in line with previous go/no-go studies (e.g., Lindemann et al., 2006). Conceptual processing demands were manipulated across experiments by presenting the words either on their own, within appropriate sentence contexts, or in conjunction with videos demonstrating the action denoted by the word. Thus, the dual task methodology used in this study was theoretically capable of and designed to optimize the likelihood of finding a necessary differential and somatotopic involvement of the motor cortices in processing words with motor-related meaning, if one exists.

## Experiment 1: single words

### Methods

#### Participants

Twenty-five (17 females, 8 males) healthy volunteers participated in this study. All were right handed and native or longstanding English speakers according to their responses on self-report measures. We did not exclude bilingual participants who acquired English as a second language (L2) early in life as the available evidence indicates these individuals have left-hemisphere cerebral language organization similar to monolinguals (see Paradis, 1990, 2006). Their ages ranged from 14 to 49 (*M* = 24.08, SD = 10.95). Informed consent was obtained from all participants.

#### Stimuli and apparatus

Stimuli consisted of eight monosyllabic, monomorphemic words 3–5 letters in length (foot, kick, hand, grab, mouth, bite, tail, wag) and “no word” baseline condition. The words were chosen such that one name and one action word related to each of four body-parts with one body-part being a non-human control that also contained concrete concepts, lexical information, yet human body irrelevant semantic content, thus ensuring a low relatedness proportion consistent with automatic meaning activation (see Neely, 1991). All words were related to only one body-part, were of medium to high frequency (>5.5 log HAL frequency; Lund and Burgess, 1996), medium to high imageability (>4.5; Cortese and Fugett, 2004), high familiarity (ratings of 6–7; Nusbaum et al., 1984), and acquired within the first 5.5 years of life (Kuperman et al., 2012). In addition, according to a corpus-based percentage measure of each word's dominant part-of-speech (PoS) relative to total frequency (Brysbaert et al., 2012), the action words were more likely to be assigned verb status (mean 79%, range 58–96) and the body-part names were more likely to be assigned noun status (97%, range 92–99).

Note that in their simplest, unmarked (i.e., infinitive) forms, English action words are ambiguous with respect to grammatical category, i.e., they may be read as either a noun or as a verb in imperative form (e.g., “kick!”; see Postle et al., 2008). If the latter, then it is possible some event structure information addressing aspects of action representation that involve the goals and intentions of agents could be accessed with verb meaning (the agent being the reader), and engage the mirror neuron system according to some embodied theories (e.g., Kemmerer and Gonzalez-Castillo, 2010). Shebani and Pulvermüller (2013) likewise employed English infinitive forms in their dual-task investigation. Like those authors, we use the term “action word” to acknowledge the grammatical ambiguity of the unmarked forms.

While completing the verbal task, participants engaged in the tapping task on a board with two buttons 3 cm in diameter and 6 cm apart (center–center). Computer software recorded the inter-tap-interval (ITI) and calculated the average time in milliseconds taken for participants to complete tapping one button and then the other while verbal stimuli were presented.

#### Procedure

All participants were tested individually. They were told that they would be required to tap alternate buttons on the tapping board as quickly and consistently as they could and that their tapping speed and not force was being measured. Although they could tap with any part of any finger, they had to move their entire finger/hand from one button to the next. They could start with either button, but could only tap with one hand at a time. They were instructed that while tapping they should repeatedly read aloud the word displayed on the computer screen for the full time while tapping. They were instructed that after the tapping task, they would be required to recall as many of the words they had read aloud during the experiment as they could. It was proposed the best way to accomplish this was to think about the word meanings as they read the words. The recall task ensured all participants attended to the different word semantics and processed all words beyond mere visual perception. More pronounced lateralized dual task decrements have been found if participants expect to be later tested on the content of the verbal task (Hellige and Longstreth, 1981).

The presentation of the 18 different tapping hand × word combinations (two hands × nine concurrent verbal tasks) were randomized and counterbalanced in order to minimize order effects. Each participant completed six sets of these 18 combinations—three sets had a randomized order of presentation and three had the reverse of these randomized sequences. Consequently, participants completed 108 trials in total.

The computer screen displayed the words “FOR THE NEXT CONDITION TAP WITH YOUR X HAND” (X being either LEFT or RIGHT), and then three 3 s later displayed “BEGIN TAPPING NOW.” After two and a half seconds (so they had established a tapping rhythm) the verbal stimuli was presented. At this point participant's ITI began being recorded in milliseconds. The stimuli were displayed for 5 s after which “STOP” was presented. At this point tapping ceased being recorded. This procedure recycled until all trials were presented. The delay between the presentations of each word (while the tapping hand and “BEGIN TAPPING NOW” instructions were displayed) was to reduce any interference from the previous word. If a participant indicated the need for a break, they completed the current trial at which point the program was paused until the participant indicated they were ready to continue. Following the final trial, participants recalled as many of the target words as they could remember.

### Results

#### Word recall results

On average each participant correctly recalled 7.32 (SD = 0.95) of the 8 target words. No one semantic or lexical category was substantially better recalled than any other semantic or lexical category.

#### Dual task results: diagnostics

There was no missing data. Outliers in the raw ITI data (cut-off = ±3 SD) were removed and each participant's mean and standard deviation ITI for each of the 18 conditions was calculated from the raw ITI data. Checking the z-scores (cut-off = ±3.29) and all possible bivariate scatterplots for this final data set revealed no univariate or bivariate outliers. Each variable's skew, kurtosis (cut-offs = ±3.29) and histograms indicated normality. The sphericity assumption was violated for several omnibus tests, however, assuming sphericity for these analyses did not produce different outcomes from those obtained using Greenhouse–Geisser, Huynh–Feldt, and lower-bound epsilon adjustments. As such all analyses were run and interpreted as if the sphericity assumption was met. An alpha level of 0.05 was used for all statistical tests, with Bonferroni and Helmert procedures used and noted where appropriate.

#### 2 × 3 (tapping hand × reading condition) ANOVA

A 2 × 3 factorial repeated measures ANOVA was conducted to assess the base lateralized dual task decrement. The variables in this analysis were: tapping hand with two levels (right vs. left); and reading condition with three levels (no reading, reading body-part names, reading action words). This analysis indicated a significant main effect of tapping hand, *F*_(1, 24)_ = 88.78, *p* < 0.001, MSE = 119.87, part-η^2^ = 0.79, such that left hand tapping was significantly slower (*M* = 242.14, SD = 28.84) than right hand tapping (*M* = 226.60, SD = 31.67). There was also a significant main effect of reading condition, *F*_(2, 48)_ = 6.11, *p* = 0.004, MSE = 56.79, part-η^2^ = 0.20. When further analyzed by Helmert linear contrasts to control family wise error (α = 0.05), this main effect indicated that participants tapped significantly slower while reading any words (*M* = 234.86, SD = 30.37) than while not reading (*M* = 230.48, SD = 27.90), *F*_(1, 24)_ = 6.33, *p* = 0.019, MSE = 75.65, part-η^2^ = 0.21, and slower while reading body-part names (*M* = 235.60, SD = 30.76) than while reading action words (*M* = 234.12, SD = 30.07), *F*_(1, 24)_ = 4.30, *p* = 0.049, MSE = 12.71, part-η^2^ = 0.15.

**Figure 1:**
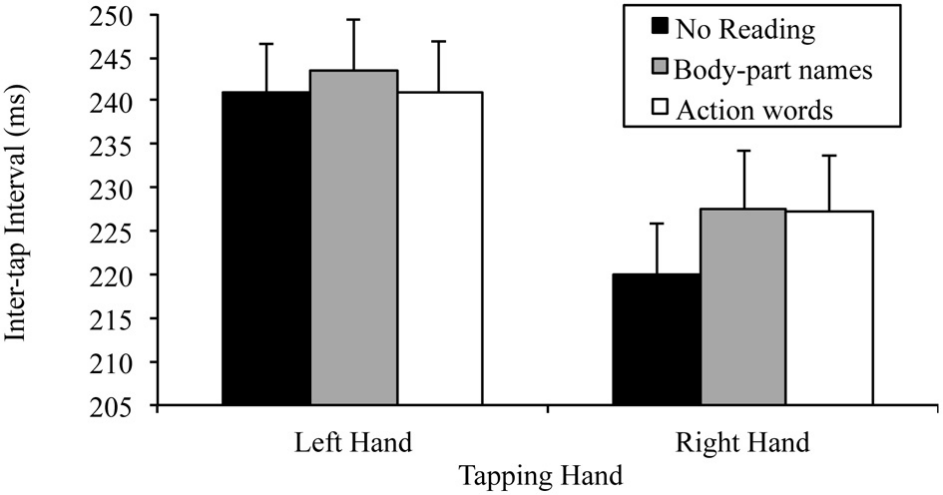
**Mean inter-tap intervals of each hand under the different reading conditions (error bars represent the standard error of the mean)**.

There was also a significant tapping hand × reading condition interaction for this 2 × 3 ANOVA, *F*_(2, 48)_ = 6.81, *p* = 0.002, MSE = 22.98, part-η^2^ = 0.22. This interaction was further examined by comparing the effects of reading condition on the right and left hand separately. The tests for the simple effects of reading condition at the two levels of tapping hand indicated, as predicted, no effect of reading on left hand tapping, *F*_(2, 48)_ = 1.78, *p* = 0.180, MSE = 32.91, part-η^2^ = 0.69, indicating no difference between the tapping speed of the left hand regardless of whether there was no reading (*M* = 240.85, SD = 28.63), body-part names read (*M* = 243.58, SD = 29.42) or action words read (*M* = 241.03, SD = 28.79). There was, however, a significant effect of reading condition on right hand tapping, *F*_(2, 48)_ = 9.50, *p* < 0.001, MSE = 46.85, part-η^2^ = 0.28. Simple comparisons using a Bonferroni correction to control family wise error (α = 0.05), indicated that, as predicted, right hand tapping was significantly slower while reading body-part names (*M* = 227.61, SD = 32.70) or action words (*M* = 227.20, SD = 31.96) than while not reading (*M* = 220.11, SD = 28.62), *t*_(24, 3 comparisons)_ = −3.23, *p* = 0.011, *d* = 0.65; *t*_(24, 3 comparisons)_ = −3.20, *p* = 0.011, *d* = 0.64, respectively. However, there was no difference in right hand tapping speed between reading body-part names (*M* = 227.61, SD = 32.70) and action words (*M* = 227.20, SD = 31.96), *t*_(24, 3 comparisons)_ = 0.42, *p* > 0.999, *d* = 0.08. Thus, the base lateralized dual task decrement was found. Figure 1 summarizes these results.

#### 2 × 8 (tapping hand × word read) ANOVA

**Figure 2:**
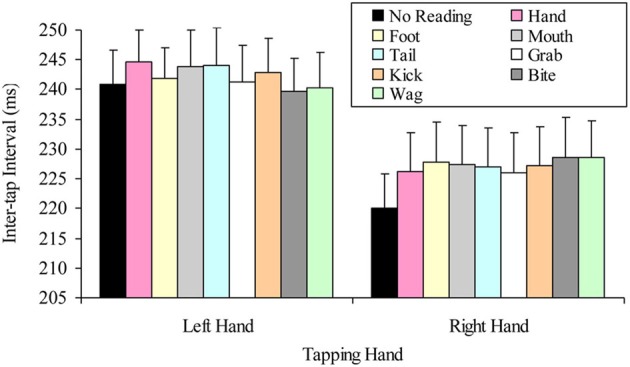
**Mean inter-tap intervals of each hand while reading the different words (error bars represent the standard error of the mean)**.

The question of whether the right hand lateralized dual task decrement would be more pronounced when participants read hand-related words (compared to other words), was tested by a 2 × 8 factorial repeated measures ANOVA. The variables in this analysis were: tapping hand with two levels (right vs. left); and word read with eight levels (hand, grab, foot, kick, mouth, bite, tail, and wag). This analysis indicated a significant main effect of tapping hand *F*_(1, 24)_ = 72.53, *p* < 0.001, MSE = 306.06, part-η^2^ = 0.75, such that left hand tapping was significantly slower (*M* = 242.14, SD = 28.84) than right hand tapping (*M* = 226.60, SD = 31.67). However, the main effect of word read was not significant, *F*_(7, 168)_ = 0.98, *p* = 0.445, MSE = 39.75, part-η^2^ = 0.04, indicating no difference in tapping speed while reading the different words. The interaction was also not significant, *F*_(7, 168)_ = 1.52, *p* = 0.165, MSE = 39.14, part-η^2^ = 0.06. Figure 2 summarizes these results.

#### 3 × 2 × 9 (trial set × tapping hand ×word read) ANOVA

Given that Medland et al. (2002) found that the lateralized dual task decrement decreased with practice, a 3 × 2 × 9 factorial repeated measures ANOVA was conducted to test for any practice effects. The variables in this analysis were: trial set with three levels (first, second, and third set of randomly generated forward and reverse presentations of condition trials); tapping hand with two levels (right vs. left); and word read with nine levels (no reading, reading of “hand,” “grab,” “foot,” “kick,” “mouth,” “bite,” “tail,” or “wag”). However, no significant differential practice effect was found as evidenced by the three way interaction being non-significant, *F*_(16, 384)_ = 0.90, *p* = 0.565, MSE = 120.49, part-η^2^ = 0.04. That is, right and left hand tapping speed while reading the different words did not vary across the three sets of trials.

### Discussion

The base lateralized dual task decrement was found; performing word reading concurrently with hand tapping differentially reduced right hand tapping rate. Despite this effect, individual word effects were not found. Although lexical category (action words vs. body-part names) was found to influence overall tapping rates, this effect was not significant for the tapping rates of either hand. That is, right hand motor performance did not differ according to the semantic or lexical category of word being read. This cannot be attributed to practice effects as the lateralized dual task decrement did not diminish with practice. That is, while motor performance became faster across all conditions as the experiment progressed (as would be expected), right and left hand motor performance during the reading task did not differ between the three sets of trials. Three possible explanations exist for these null results.

The first potential explanation for these null results relates to the degree of complexity of the stimuli. Specifically, it is possible that a differential dual task effect was not found for hand-related words (action words and body-part names), as the verbal stimuli were simple one-syllable words and thus not sufficiently cognitively demanding to differentially activate the motor areas. For example, Ashton and McFarland (1991) found the dual task decrement to be more pronounced for right hand tapping while reciting a tongue twister (high cognitive demands) compared to reciting single phonemes (e.g., “la-la”; low cognitive demands). Therefore, it is possible that simplicity of the stimuli resulted in a less pronounced dual task effect, masking any differential dual task effect for the different words. However, it is worth noting the majority of go/no-go studies have employed single words (e.g., Lindemann et al., 2006; Sato et al., 2008; Mirabella et al., 2012).

The second potential explanation for the null results is also associated with the relative simplicity of the verbal stimuli. Specifically, evidence indicates that prolonged inspection and/or repetition of a word can temporarily block access to the word's meaning, resulting in the subjective experience of decreased word meaningfulness (Esposito and Pelton, 1971; Smith and Klein, 1990; Frenck-Mestre et al., 1997; Black, 2001). This effect, commonly referred to as semantic satiation, may have occurred in this experiment and would have resulted in the target words not being readily associated with their meanings. Although participants recalled the stimuli in this experiment, it could be that repeated exposure to the simple stimuli rather than processing of word meaning was responsible for the high recall rate. Again, it is worth noting that the majority of go/no-go studies have likewise employed multiple repetitions of single words (e.g., Lindemann et al., 2006; Sato et al., 2008; Mirabella et al., 2012).

The third potential explanation for the null results is that having participants read the words aloud entailed articulatory-motor movements, and the associated motor system activity may have “over-ridden” any meaning related activity differences. Reading silently has been found to produce less pronounced dual task decrements than reading aloud (Bowers et al., 1978; Hellige and Longstreth, 1981; Medland et al., 2002). However, as the motor task involved hand tapping, such an explanation would not be consistent with claims regarding a motor semantic somatotopy.

Finally, it is also possible that the non-significant result for hand related words reflects the *absence* of a motor semantic somatotopy.

## Experiment 2: sentence context and reading aloud vs. silently

Experiment 2 was conducted to test whether the null results from Experiment 1 were due to the stimuli being too simple via manipulating the complexity of the verbal task. This was to ensure that participants processed the target words and associated them with the actions and body-parts they describe. Despite evidence indicating simple cognitive tasks reduce the strength of the dual task effect (e.g., Ashton and McFarland, 1991), the verbal task cannot be made too demanding as evidence also indicates that the dual task decrement is *reduced* when concurrent cognitive tasks are *overly* demanding (e.g., McFarland and Ashton, 1978a,b). As such, Experiment 1 was repeated with the target words embedded in sentences differing in syntactic and phonological complexity. Embedding the target words in sentence contexts was also likely to making their meanings more clear and reduce effects of semantic satiation.

Chomsky (1957) proposed the theory of transformational grammar, which suggested that every sentence has two structures: surface structure representing the arrangement and choice of words; and deep structure representing the sentence meaning. He proposed that to extract a sentence's meaning the brain transforms the surface structure to make it reflect the deep structure. Kernel sentences, or “declarative and active (sentences) with no complex verb or noun phrases” (Chomsky, 1957; p. 107) and no phonological sequences that are difficult to read, more closely reflect the deep structure than other sentences. As such, they require no transformations and thus less cognitive effort to extract the sentence's meaning. However, reading these sentences would still involve more cognitive effort than reading single words. Consequently, this was used as the definition of a simple sentence.

Syntactic transformations can be applied to kernel sentences to make them more syntactically complex and cognitively demanding without altering the deep structure (Chomsky, 1957). For instance, passive transformations of kernel sentences have the same deep structure but are more syntactically complex as they alter the surface structure from being the more common (in English) subject-verb-object to the less common (in English) object-verb-subject. Thus, the sentence must be rearranged (transformed) to extract the deep structure—a process involving cognitive effort. This is supported by evidence suggesting that passive sentences take longer to read and process, are more attention demanding (Miller, 1962; Britton et al., 1982; Clifton and Duffy, 2001) yet do not differ in comprehension accuracy (Bradley and Meeds, 2002) when compared to the active sentences from which they were derived. However, passive transformations add words to the kernel sentence and can change the tense, which may detract from the content of the target words. Thus, this experiment used a passive like transformation as the definition for syntactic complexity requiring the sentence to be rearranged to extract the deep structure (thereby involving more cognitive effort than reading the simple kernel sentence) while keeping the number of words and tense constant across the transformation.

Making a sentence phonologically complex can also increase the cognitive demand associated with processing it. Phonological complexity is best represented by tongue twisters, or sentences where the majority of words have the same initial phoneme (McCutchen and Perfetti, 1982). When processing tongue twisters, many key words with the same initial phoneme must be substituted with synonyms with a different initial phoneme to reduce articulation demands and make the sentence more easily reflect the deep structure, a process involving cognitive effort. That tongue twisters require greater cognitive effort is demonstrated by their taking longer to read (aloud or silently), involving more recall errors and less accurate semantic judgments than phonologically simple sentences with the same deep structure (McCutchen and Perfetti, 1982; Hanson et al., 1991; McCutchen et al., 1991; Zhang and Perfetti, 1993; Keller et al., 2003). Furthermore, Ashton and McFarland (1991) found greater right hand tapping dual task interference when participants recited tongue twisters compared to single phonemes. Therefore, this experiment used tongue twisters to manipulate phonological complexity.

In summary, compared to simple sentences, passive sentences involve additional cognitive processing though do not affect sentence comprehension, while phonologically complex sentences (tongue twisters) require additional cognitive processing and do influence sentence comprehension. Consequently, if sentence complexity moderates body-part related word comprehension, this manipulation should elicit differential right hand tapping rates for hand related sentences compared to the other stimuli. Finally, Experiment 2 also included a between-groups manipulation of reading aloud vs. silently, to determine whether articulatory-motor movements might have obscured any dual-task differences in Experiment 1.

### Methods

#### Participants

Fifty (31 females, 19 males) healthy volunteers participated in this study. Their ages ranged from 15 to 48 (*M* = 21.98, SD = 6.40). Informed consent was obtained from all participants. All were right handed and native or longstanding English speakers. The 50 participants were randomly allocated to one of two groups—reading aloud or silently. The reading aloud group consisted of 20 participants (13 females, 7 males) whose ages ranged from 15 to 34 (*M* = 20.25, SD = 3.97). The reading silently group consisted of 30 participants (18 females, 12 males). Their ages ranged from 17 to 48 (*M* = 23.13, SD = 7.45).

#### Stimuli and apparatus

These were identical to Experiment 1, however, each of the eight target words were embedded in simple, syntactically complex or phonologically complex sentences (24 sentences in total). Tense, perspective, serial position of the target word, and number of syllables and words was constant and the sentences put the target words in a context likely to evoke strong associations with the body-part/action. Furthermore, the tongue twister remained as such when the key noun/verb was substituted for the target words (see Table 1). Including the no reading condition brought the total number of conditions performed with each hand to 25.

**Table 1:** **Sentence stimuli of Experiment 2**.

**Simple sentences**	**Syntactically complex sentences**	**Phonologically complex sentences**
Your hand is on the desk.	On the desk is your hand.	The big black hand bled blood.
Your foot is on the floor.	On the floor is your foot.	The big black foot bled blood.
Your mouth is near the ceiling.	Near the ceiling is your mouth.	The big black mouth bled blood.
The tail is at the end.	At the end is the tail.	The big black tail bled blood.
You grab objects off the table.	Off the table you grab objects.	The big black bears grab blood.
You kick objects across the field.	Across the field you kick objects.	The big black bears kick blood.
You bite objects into two parts.	Into two parts you bite objects.	The big black bears bite blood.
They wag at objects on the floor.	On the floor they wag at objects.	The big black bears wag blood.

#### Procedure and design

The procedure replicated that of Experiment 1 with the addition of a between-groups independent variable (reading aloud vs. silently). The presentation of the 50 different tapping hand × verbal task combinations (two hands × 25 verbal conditions including no reading baseline) were randomized and counterbalanced in order to minimize order effects. Each participant completed two sets of these 50 combinations—one randomized order of presentation and one the reverse of this randomized sequences. Consequently, participants completed 100 trials in total.

### Results

#### Sentence recall results

On average each participant correctly recalled 14.64 (SD = 4.69) of the 24 sentences. There was no significant difference in the number of sentences recalled between the reading aloud (*M* = 15.55, SD = 5.06) and reading silently groups (*M* = 14.03, SD = 4.41), *t*_(48)_ = 1.12, *p* = 0.267. Furthermore, no one semantic, lexical or complexity category of sentences was substantially better recalled than any other.

#### Dual task results: diagnostics

There was no missing data. Outliers and violations of sphericity were treated identically to Experiment 1. Initially the data and hypotheses were analyzed as two separate data sets (reading aloud ITIs and reading silently ITIs). These data sets were then combined and the analyses re-run on this one larger data set to increase sample size, and thus power.

#### Reading aloud: 2 × 3 (tapping hand × reading condition) ANOVA

A 2 × 3 factorial repeated measures ANOVA was conducted to assess the base lateralized dual task decrement, from which all other predictions were derived. The variables in this analysis of the reading aloud data were: tapping hand with two levels (right vs. left); and reading condition with three levels (no reading, reading aloud sentences containing body-part names, reading aloud sentences containing action words). This analysis indicated a significant main effect of tapping hand, *F*_(1, 19)_ = 35.77, *p* < 0.001, MSE = 148.94, part-η^2^ = 0.65, such that left hand tapping was significantly slower (*M* = 251.23, SD = 40.27) than right hand tapping (*M* = 237.91, SD = 44.05). There was also a significant main effect of reading condition, *F*_(2, 38)_ = 9.46, *p* < 0.001, MSE = 80.57, part-η^2^ = 0.33. When further analyzed by Helmert linear contrasts to control family wise error (α = 0.05), this main effect indicated that participants tapped significantly slower while reading aloud any sentences (*M* = 247.09, SD = 41.75) than while not reading (*M* = 239.54, SD = 47.83), *F*_(1, 19)_ = 10.32, *p* = 0.005, MSE = 110.37, part-η^2^ = 0.35, however, no tapping speed differences existed between reading aloud sentences containing body-part names (*M* = 246.83, SD = 41.47) and reading aloud sentences containing action words (*M* = 247.34, SD = 42.02), *F*_(1, 19)_ = 0.39, *p* = 0.542, MSE = 3.38, part-η^2^ = 0.02.

**Figure 3:**
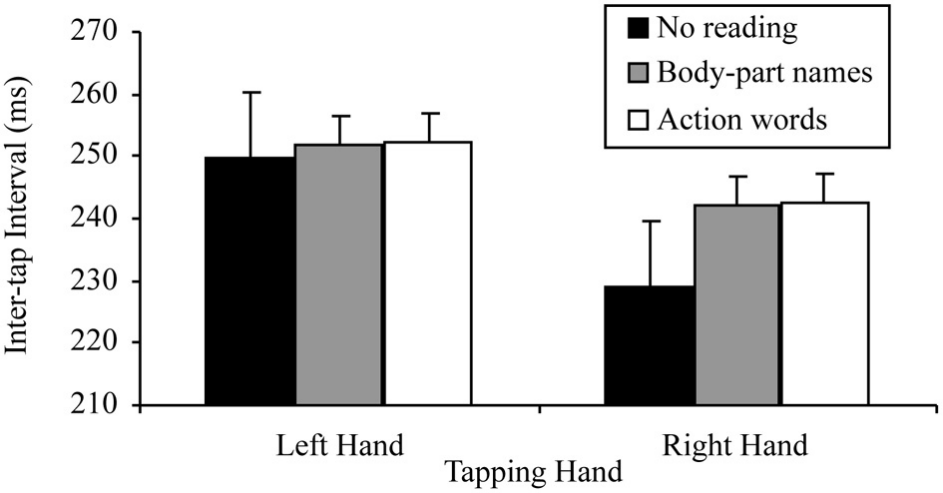
**Mean inter-tap intervals of each hand under the different reading conditions in the reading aloud data set (error bars represent one standard error of the mean)**.

There was also a significant tapping hand × reading condition interaction for this 2 × 3 ANOVA, *F*_(2, 38)_ = 5.78, *p* = 0.006, MSE = 70.34, part-η^2^ = 0.23. This interaction was further examined by comparing the effects of reading condition on the right and left hand separately. The tests for the simple effects of reading condition at the two levels of tapping hand indicated, as predicted, no effect of reading on left hand tapping, *F*_(2, 38)_ = 0.65, *p* = 0.530, MSE = 44.25, part-η^2^ = 0.03, suggesting no difference between the tapping speed of the left hand regardless of whether there was no reading (*M* = 249.88, SD = 47.96), reading aloud of sentences containing body-part names (*M* = 351.66, SD = 39.89) or reading aloud of sentences containing action words (*M* = 252.15, SD = 40.28). There was, however, a significant effect of reading condition on right hand tapping, *F*_(2, 38)_ = 10.69, *p* < 0.001, MSE = 106.67, part-η^2^ = 0.36. Simple comparisons using a Bonferroni correction to control family wise error (α = 0.05), indicated that, as predicted, right hand tapping was significantly slower while reading aloud sentences containing body-part names (*M* = 241.99, SD = 43.74) or action words (*M* = 242.54, SD = 44.18) than while not reading (*M* = 229.19, SD = 49.56), *t*_(19, 3 comparisons)_ = −3.19, *p* = 0.005, *d* = 0.71; *t*_(19, 3 comparisons)_ = −3.37, *p* = 0.003, *d* = 0.75, respectively. However, there was no difference in tapping speed between reading aloud sentences containing body-part names (*M* = 241.99, SD = 43.74) and action words (*M* = 242.54, SD = 44.18), *t*_(19, 3 comparisons)_ = −1.11, *p* = 0.281, *d* = 0.24. Figure 3 summarizes these results.

#### Reading aloud: 2 × 3 × 8 (tapping hand × sentence complexity × target word) ANOVA

The question of whether the right hand lateralized dual task decrement would be more pronounced when participants read sentences (especially simple sentences) containing hand-related words (compared to other words), was tested by a 2 × 3 × 8 factorial repeated measures ANOVA. The variables in this analysis of the reading aloud data were: tapping hand with two levels (right vs. left); sentence complexity with three levels (simple vs. syntactically complex vs. phonologically complex); and target word embedded in the sentence with eight levels (hand, grab, foot, kick, mouth, bite, tail, and wag). This analysis indicated a significant main effect of tapping hand *F*_(1, 19)_ = 18.33, *p* < 0.001, MSE = 1218.02, part-η^2^ = 0.49, such that left hand tapping was significantly slower (*M* = 251.23, SD = 40.27) than right hand tapping (*M* = 237.91, SD = 44.05). However, the main effect of sentence complexity was not significant, *F*_(2, 38)_ = 0.39, *p* = 0.638, MSE = 204.46, part-η^2^ = 0.02, suggesting no difference in tapping speed while reading aloud sentences differing in syntactic and phonological complexity. The main effect of target word was also not significant, *F*_(7, 133)_ = 0.32, *p* = 0.942, MSE = 172.82, part-η^2^ = 0.02, suggesting no difference in tapping speed while reading aloud sentences containing semantically different target words.

**Figure 4:**
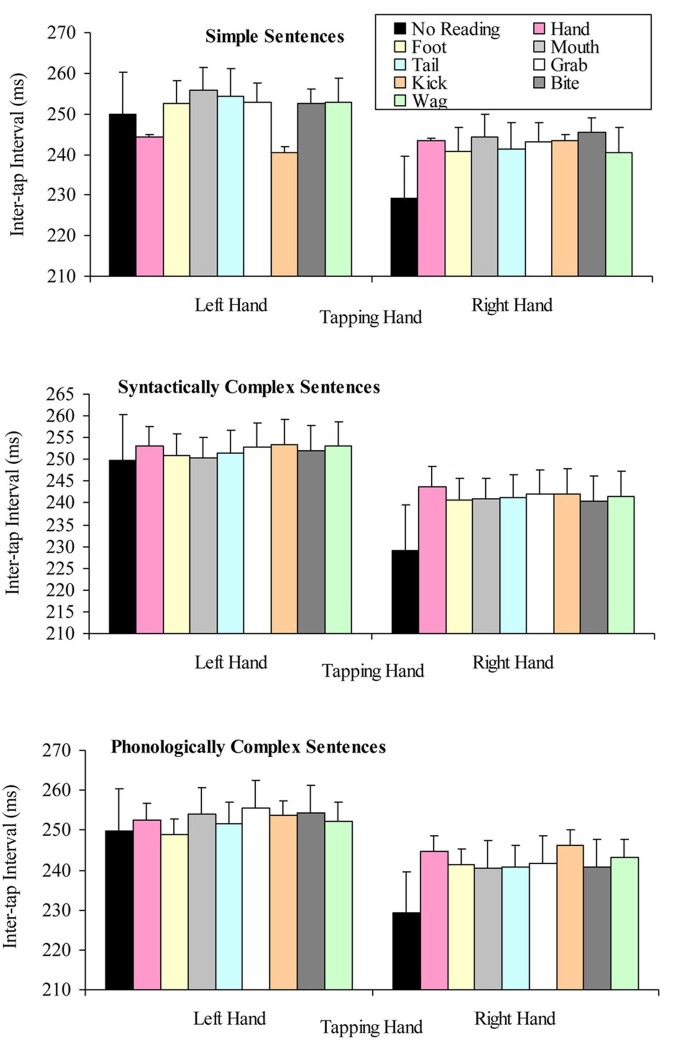
**Mean inter-tap intervals for the tapping hand × target word conditions for each type of sentence complexity in the reading aloud data set (error bars represent the standard error of the mean)**.

None of the interactions of this analysis were significant. Specifically, the tapping hand × sentence complexity interaction *F*_(2, 38)_ = 1.12, *p* = 0.336, MSE = 184.14, part-η^2^ = 0.06, tapping hand × target word interaction *F*_(7, 133)_ = 1.25, *p* = 0.278, MSE = 152.66, part-η^2^ = 06, the sentence complexity × target word interaction *F*_(14, 266)_ = 0.96, *p* = 0.501, MSE = 188.85, part-η^2^ = 0.05, and tapping hand × sentence complexity × target word interaction *F*_(14, 266)_ = 0.80, *p* = 0.667, MSE = 151.22, part-η^2^ = 0.04. Figure 4 summarizes these results.

#### Reading silently: 2 × 3 (tapping hand × reading condition) ANOVA

A 2 × 3 factorial repeated measures ANOVA was conducted to assess the base lateralized dual task decrement. The variables in this analysis of the reading silently data were: tapping hand with two levels (right vs. left); and reading condition with three levels (no reading, silently reading sentences containing body-part names, silently reading sentences containing action words). This analysis indicated a significant main effect of tapping hand, *F*_(1, 29)_ = 37.95, *p* < 0.001, MSE = 140.41, part-η^2^ = 0.59, such that left hand tapping was slower (*M* = 271.78, SD = 47.84) than right hand tapping (*M* = 263.93, SD = 52.08). However, the main effect of reading condition was not significant, *F*_(2, 58)_ = 3.72, *p* = 0.060, MSE = 88.17, part-η^2^ = 0.11, suggesting no difference in tapping speed between no reading (*M* = 268.81, SD = 52.68), silently reading sentences containing body-part names (*M* = 268.11, SD = 49.44) and silently reading sentences containing action words (*M* = 267.53, SD = 50.03).

**Figure 5:**
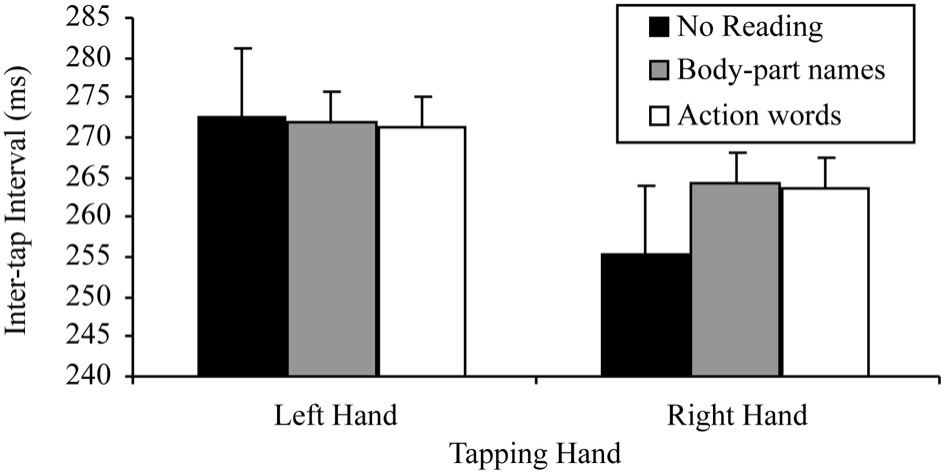
**Mean inter-tap intervals of each hand under the different reading conditions in the reading silently data set (error bars represent one standard error of the mean)**.

There was also a significant tapping hand × reading condition interaction for this 2 × 3 ANOVA, *F*_(2, 58)_ = 4.10, *p* = 0.022, MSE = 112.42, part-η^2^ = 0.12. This interaction was further examined by comparing the effects of reading condition on the right and left hand separately. The tests for the simple effects of reading condition at the two levels of tapping hand indicated, as predicted, no effect of reading on left hand tapping, *F*_(2, 58)_ = 0.08, *p* = 0.920, MSE = 107.99, part-η^2^ < 0.01, suggesting no difference between the tapping speed of the left hand regardless of whether there was no reading (*M* = 272.44, SD = 48.04), silent reading of sentences containing body-part names (*M* = 271.97, SD = 47.52) or silent reading of sentences containing action words (*M* = 271.35, SD = 47.95). There was, however, a significant effect of reading condition on right hand tapping, *F*_(2, 58)_ = 8.42, *p* < 0.001, MSE = 92.59, part-η^2^ = 0.23. Simple comparisons using a Bonferroni correction to control family wise error (α = 0.05), indicated that, as predicted, right hand tapping was significantly slower while silently reading sentences containing body-part names (*M* = 264.24, SD = 51.76) or action words (*M* = 263.71, SD = 52.61) than while not reading (*M* = 255.16, SD = 49.97), *t*_(29, 3 comparisons)_ = −3.05, *p* = 0.005, *d* = 0.56; *t*_(29, 3 comparisons)_ = −2.82, *p* = 0.009, *d* = 0.51, respectively. However, there was no difference in tapping speed between silently reading sentences containing body-part names (*M* = 264.24, SD = 51.76) and action words (*M* = 263.71, SD = 52.61), *t*_(29, 3 comparisons)_ = 0.82, *p* = 0.421, *d* = 0.18. These results are consistent with those of the reading aloud data set. Figure 5 summarizes these results.

#### Reading silently: 2 × 3 × 8 (tapping hand × sentence complexity × target word) ANOVA

The question of whether the right hand lateralized dual task decrement would be more pronounced when participants read sentences (but especially simple sentences) containing hand-related words (compared to other words), were tested by a 2 × 3 × 8 factorial repeated measures ANOVA. The variables in this analysis of the reading silently data were: tapping hand with two levels (right vs. left); sentence complexity with three levels (simple vs. syntactically complex vs. phonologically complex); and target word that the sentence contained with eight levels (hand, grab, foot, kick, mouth, bite, tail, and wag). This analysis indicated a significant main effect of tapping hand *F*_(1, 29)_ = 16.54, *p* < 0.001, MSE = 1284.51, part-η^2^ = 0.36, such that left hand tapping was significantly slower (*M* = 271.78, SD = 47.84) than right hand tapping (*M* = 263.93, SD = 52.08). However, the main effect of sentence complexity was not significant, *F*_(2, 58)_ = 1.16, *p* = 0.321, MSE = 178.71, part-η^2^ = 0.04, suggesting no difference in tapping speed while silently reading sentences differing in syntactic and phonological complexity. The main effect of target word was also not significant, *F*_(7, 203)_ = 1.07, *p* = 0.385, MSE = 120.58, part-η^2^ = 0.04, suggesting no difference in tapping speed while silently reading sentences containing semantically different target words. These results are consistent with those of the reading aloud data.

**Figure 6:**
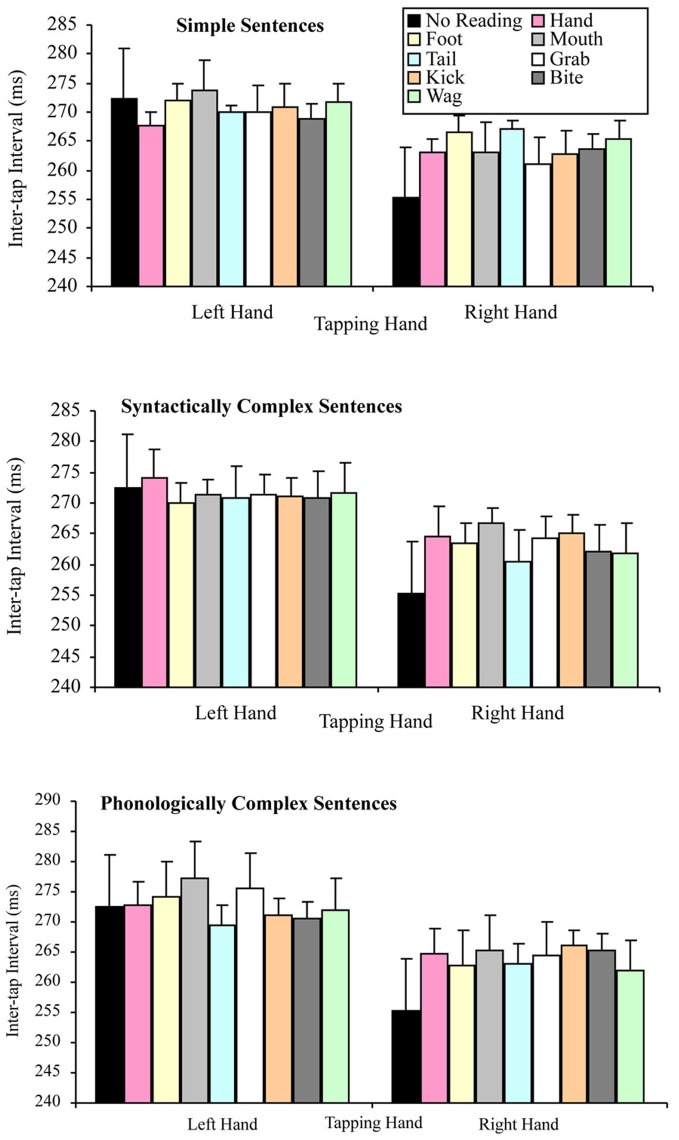
**Mean inter-tap intervals for the tapping hand × target word conditions for each type of sentence complexity in the reading silently data set (error bars represent the standard error of the mean)**.

As was the case in the reading aloud data set, none of the interactions of this analysis of the reading silently data were significant. Specifically, the tapping hand × sentence complexity interaction *F*_(2, 58)_ = 1.22, *p* = 0.302, MSE = 116.30, part-η^2^ = 0.04, the tapping hand × target word interaction *F*_(7, 203)_ = 0.70, *p* = 0.675, MSE = 94.95, part-η^2^ = 0.02, the sentence complexity × target word interaction *F*_(14, 406)_ = 1.12, *p* = 0.342, MSE = 114.74, part-η^2^ = 0.04, and the tapping hand × sentence complexity × target word interaction *F*_(14, 406)_ = 0.92, *p* = 0.539, MSE = 118.34, part-η^2^ = 0.03. Figure 6 summarizes these results.

#### Combined reading aloud and silently datasets: 2 × 3 (tapping hand × reading condition) ANOVA; and 2 × 3 × 8 (tapping hand × sentence complexity × target word) ANOVA

As there were no substantiative differences between the interpretation of the reading aloud and reading silently analyses, these two data sets were combined and all analyses rerun to increase the sample size, and thus power. These analyses revealed the same pattern of results as those found in the separate reading alone and reading silently data sets.

More specifically, a 2 × 3 factorial repeated measures ANOVA was conducted to assess the base lateralized dual task decrement. The variables in this analysis of the combined data set were: tapping hand with two levels (right vs. left); and reading condition with three levels (no reading, reading sentences containing body-part names, reading sentences containing action words). As in the separate data set analyses, this analysis indicated a significant main effect of tapping hand, *F*_(1, 49)_ = 73.75, *p* < 0.001, MSE = 143.05, part-η^2^ = 0.60, such that left hand tapping was significantly slower (*M* = 263.80, SD = 45.62) than right hand tapping (*M* = 255.05, SD = 49.78). The main effect of reading condition was also significant, *F*_(2, 98)_ = 11.49, *p* < 0.001, MSE = 85.61, part-η^2^ = 0.19. When further analyzed by Helmert linear contrasts to control family wise error (α = 0.05), this main effect indicated that participants tapped significantly slower while reading any sentences (*M* = 259.52, SD = 47.38) than while reading nothing (*M* = 257.10, SD = 52.34), *F*_(1, 49)_ = 12.25, *p* = 0.001, MSE = 120.37, part-η^2^ = 0.20, however, no tapping speed differences existed between reading sentences containing body-part names (*M* = 259.59, SD = 47.16) and reading sentences containing action words (*M* = 259.45, SD = 47.60), *F*_(1, 49)_ = 0.09, *p* = 0.762, MSE = 10.71, part-η^2^ < 0.01.

**Figure 7:**
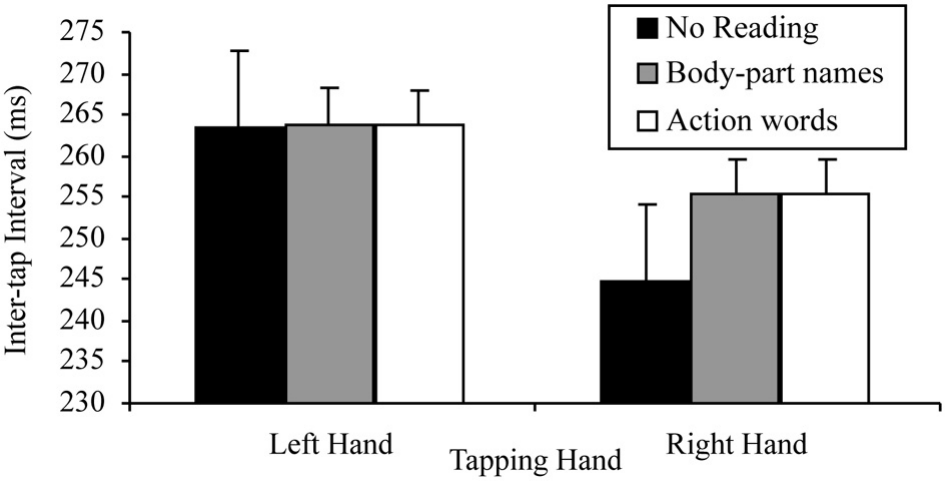
**Mean inter-tap intervals of each hand under the different reading conditions in the combined data set (error bars represent one standard error of the mean)**.

As in the separate data set analyses, there was also a significant tapping hand × reading condition interaction for this 2 × 3 ANOVA, *F*_(2, 98)_ = 9.20, *p <* 0.001, MSE = 93.90, part-η^2^ = 0.16. This interaction was further examined by comparing the effects of reading on the right and left hand separately. The tests for the simple effects of reading at the two levels of tapping hand indicated, as predicted, no effect of reading on left hand tapping, *F*_(2, 98)_ = 0.30, *p* = 0.972, MSE = 81.80, part-η^2^ < 0.01, suggesting no difference between the tapping speed of the left hand regardless of whether there was no reading (*M* = 263.42, SD = 48.81), reading of sentences containing body-part names (*M* = 263.85, SD = 45.33) or reading of sentences containing action words (*M* = 263.67, SD = 45.61). There was, however, a significant effect of reading on right hand tapping, *F*_(2, 98)_ = 18.88, *p* < 0.001, MSE = 97.71, part-η^2^ = 0.28. Simple comparisons using a Bonferroni correction to control family wise error (α = 0.05), indicated that, as predicted, right hand tapping was significantly slower while reading sentences containing body-part names (*M* = 255.34, SD = 49.49) or action words (*M* = 255.24, SD = 50.05) than while not reading (*M* = 244.77, SD = 50.94), *t*_(49, 3 comparisons)_ = −4.43, *p* < 0.001, *d* = 0.64; *t*_(49, 3 comparisons)_ = −4.34, *p* < 0.001, *d* = 0.61, respectively. However, there was no difference in tapping speed between reading sentences containing body-part names (*M* = 255.34, SD = 49.49) and action words (*M* = 255.24, SD = 50.05), *t*_(49, 3 comparisons)_ = 0.23, *p* = 0.816, *d* = 0.03. Figure 7 summarizes these results.

The question of whether the right hand lateralized dual task decrement would be more pronounced when participants read sentences (but especially simple sentences) containing hand-related words (compared to other words), were tested by a 2 × 3 × 8 factorial repeated measures ANOVA. The variables in this analysis of the combined data set were: tapping hand with two levels (right vs. left); sentence complexity with three levels (simple vs. syntactically complex vs. phonologically complex); and target word that the sentence contained with eight levels (hand, grab, foot, kick, mouth, bite, tail, and wag). As in the separate data set analyses, this analysis indicated a significant main effect of tapping hand *F*_(1, 49)_ = 34.58, *p* < 0.001, MSE = 1243.84, part-η^2^ = 0.41, such that left hand tapping was significantly slower (*M* = 263.80, SD = 45.62) than right hand tapping (*M* = 255.05, SD = 49.78). However, the main effect of sentence complexity was not significant, *F*_(2, 98)_ = 1.52, *p* = 0.224, MSE = 185.15, part-η^2^ = 0.03, suggesting no difference in tapping speed while reading sentences differing in syntactic and phonological complexity. The main effect of target word was also not significant, *F*_(7, 343)_ = 0.73, *p* = 0.647, MSE = 140.07, part-η^2^ = 0.02, suggesting no difference in tapping speed while reading sentences containing the semantically different target words.

**Figure 8:**
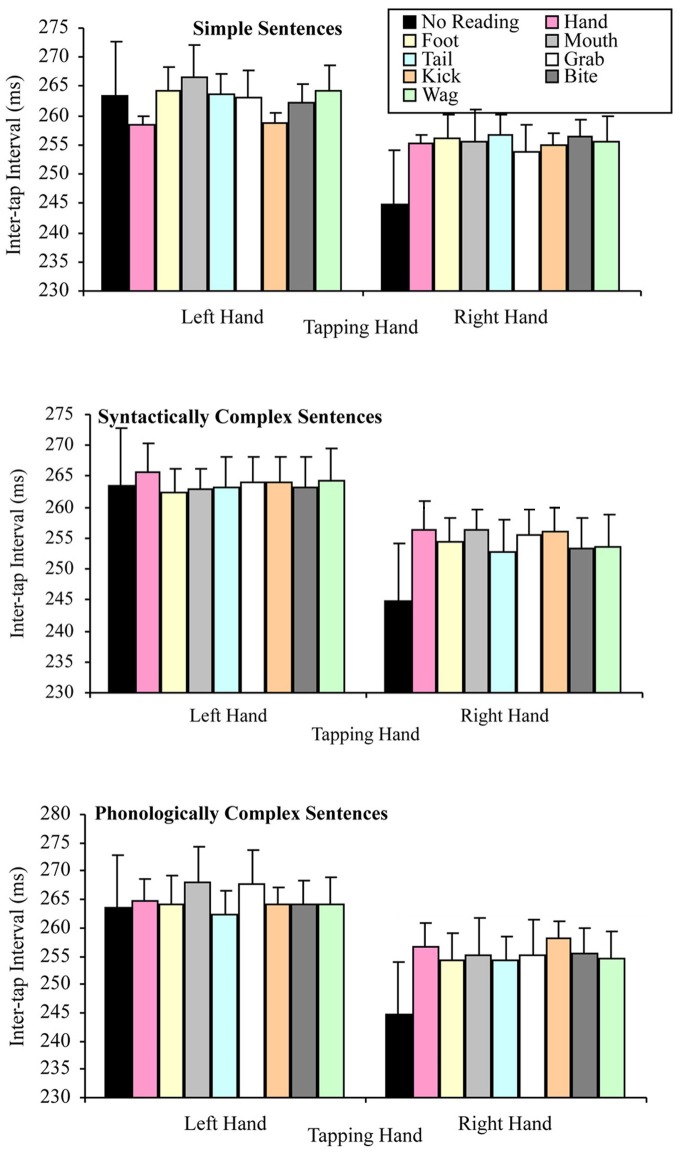
**Mean inter-tap intervals for the tapping hand × target word conditions for each type of sentence complexity in the combined data set (error bars represent the standard error of the mean)**.

As in the separate data set analyses, none of the interactions in the combined data were significant. Specifically, the tapping hand × sentence complexity interaction, *F*_(2, 98)_ = 2.26, *p* = 0.110, MSE = 132.91, part-η^2^ = 0.04, the tapping hand × target word interaction, *F*_(7, 343)_ = 1.43, *p* = 0.191, MSE = 117.22, part-η^2^ = 0.03, the sentence complexity × target word interaction, *F*_(14, 686)_ = 1.40, *p* = 0.148, MSE = 143.33, part-η^2^ = 0.03, the tapping hand × sentence complexity × target word interaction, *F*_(14, 686)_ = 0.71, *p* = 0.768, MSE = 131.47, part-η^2^ = 0.01. Figure 8 summarizes these results.

### Discussion

Experiment 2 was designed to test whether the null results from Experiment 1 were due to the simplicity of the verbal stimuli and/or the presence of motor-articulation obscuring effects. As in Experiment 1, the base lateralized dual task decrement from was found for both reading aloud and silently. Despite the base effect being found for both reading aloud and reading silently, individual word effects were not found for any of the three data sets (reading aloud, reading silently, and combined). That is, right hand motor performance did not differ according to the semantic or lexical category of word being read. This is also consistent with the findings of Experiment 1.

One possible explanation of the null results of Experiment 1 was that the verbal stimuli were not sufficiently cognitively demanding, resulting in a less pronounced overall dual task decrement. However, no support was found for this explanation. This experiment manipulated the complexity of the verbal stimuli to assess this possibility, however, no individual word effects were found for any of the sentences differing in complexity. While the dual task decrement can be reduced when concurrent cognitive tasks are too demanding (McFarland and Ashton, 1978a,b), this is unlikely to have occurred here as the base lateralized dual task decrement was found for all three data sets and was found to be equally pronounced across all levels of sentence complexity, suggesting the capability of finding individual word effects.

Another possible explanation of the null results of Experiment 1 was semantic satiation, whereby prolonged inspection and repetition of a word can temporarily block access to the word's meaning, resulting in the target words not being associated with the actions and body-parts they describe (Esposito and Pelton, 1971; Smith and Klein, 1990; Frenck-Mestre et al., 1997; Black, 2001). This experiment embedded the target words in sentences to minimize prolonged inspection and repetition of the target words alone. As no individual word effects were found despite these changes in stimuli, this suggests that semantic satiation was unlikely to have occurred within this data and as such is an unlikely explanation for the null results.

That participants may not have associated the target words with the action/body-part they described is still a possibility, despite the sentences embedding the words in context. Furthermore, it might be claimed that the complexity manipulation may have had the *reverse* effect to that intended, i.e., participants may not have been focusing their attention on the target word in the sentences due to the complexity manipulation, and may have instead directed attention to phrasal level variables such as word ordering. Therefore, it is possible that the association between the target words and the relevant action and body-part in this experiment was still not strong enough to elicit the expected differential dual task effects.

## Experiment 3: sentences and videos

Experiment 3 aimed to replicate Experiments 1 and 2 with the addition of videos of the actions and body-parts described by the target words while participants read only the simple sentences from Experiment 2. While viewing these videos would likely involve spatial processing and thus elicit right hemisphere activity, it was expected that these videos would elicit visuomotor associations linked to the meaning of the target words (the actions and body-parts described) present in the context of the experiment, which in turn should elicit semantic motor cortex activity. This expectation was based on evidence interpreted as supporting “mirror” visuomotor neurons in the human motor cortices that respond when an action is executed and observed (e.g., Koski et al., 2002; Lamm et al., 2007; Postle et al., 2008), and the proposal that these mirror neurons may code action at an abstract level that is accessible by language (Gallese and Lakoff, 2005; Rizzolatti and Craighero, 2004). Therefore, processing action words in the context of viewing the actions described should facilitate the involvement of the mirror neuron system and thus elicit somatotopic motor cortex activity during the reading of action and body-part related words.

### Methods

#### Participants

Twenty-five (19 females, 6 males) healthy volunteers participated in this study. All were right handed and native or longstanding English speakers according to their responses on self-report measures. Their ages ranged from 18 to 33 (*M* = 23.88, SD = 3.88). Informed consent was obtained from all participants.

#### Stimuli and apparatus

This experiment used the eight simple sentences from Experiment 2 and five videos each of 5 s duration with no audio track, in addition to a tapping only baseline (i.e., 14 conditions to be performed with each hand). Four of the videos depicted the actions described by the target action words being repeatedly performed (a hand performing a grabbing action, a foot and lower leg performing a kicking action, a mouth and lower face performing a biting action, and a dog's tail performing a wagging action) and one depicting a movement unrelated to the body (water moving in a fountain). No other stimuli were present in the frame to ensure all attention was directed to the body-part and the action being performed. The hand movement video depicted a right hand as we wished to elicit visuomotor associations within the language-dominant left hemisphere, consistent with both the proposed role/mechanism for mirror neurons in language comprehension and the mechanism responsible for the lateralized dual-task decrement. As movement interference occurs when participants concurrently observe and execute incongruent or incompatible actions with the same hand (e.g., Kilner et al., 2003), and is proposed to be due to co-activation of conflicting populations of mirror neurons, we did not employ a video of a left hand performing a grabbing movement (as this would be likely to elicit interference in the left-hand tapping condition, unrelated to the left-hemisphere cerebral organization of language and right hand preference).

#### Procedure and design

The procedure was similar to those of Experiment 1 and 2. The presentation of the 28 different tapping hand × task combinations (two hands × 14 stimuli) were randomized and counterbalanced in order to minimize order effects. Each participate completed four sets of these 28 combinations—two randomized order of presentation and two the reverse of these randomized sequences. Consequently, participants completed 112 trials in total. The sentences were read aloud and the videos were passively viewed.

### Results

#### Sentence recall results

On average each participant correctly recalled 11.56 (SD = 1.50) of the 13 stimuli (eight sentences and five videos). For the video stimuli, 24 of the 25 participants recalled all five videos, with only one participant failing to recall the mouth video. On average, each participant correctly recalled 6.64 (SD = 1.44) of the eight sentences. No one semantic or lexical category was substantially better recalled than any other.

#### Dual task results: diagnostics

There was no missing data. Outliers and violations of sphericity were treated identically to Experiment 1 and 2. An alpha level of 0.05 was used for all statistical tests, with Bonferroni and Helmert procedures used and noted where appropriate.

#### 2 × 4 (tapping hand × concurrent task) ANOVA

A 2 × 4 factorial repeated measures ANOVA was conducted to assess the base lateralized dual task decrement, from which all other predictions were derived. The variables in this analysis were: tapping hand with two levels (right vs. left); and concurrent task with four levels (no concurrent task, reading sentences containing body-part names, reading sentences containing action words, passively viewing videos). This analysis indicated a significant main effect of tapping hand, *F*_(1, 24)_ = 21.79, *p* < 0.001, MSE = 539.98, part-η^2^ = 0.48, such that left hand tapping was significantly slower (*M* = 250.37, SD = 41.71) than right hand tapping (*M* = 236.38, SD = 45.50). However, the main effect of concurrent task was not significant, *F*_(3, 72)_ = 2.56, *p* = 0.062, MSE = 62.11, part-η^2^ = 0.10, suggesting no difference in tapping speed between no concurrent task (*M* = 238.73, SD = 43.50), reading sentences containing body-part names (*M* = 242.90, SD = 42.30), reading sentences containing action words (*M* = 243.51, SD = 42.12) and passively viewing videos (*M* = 244.58, SD = 44.23).

**Figure 9:**
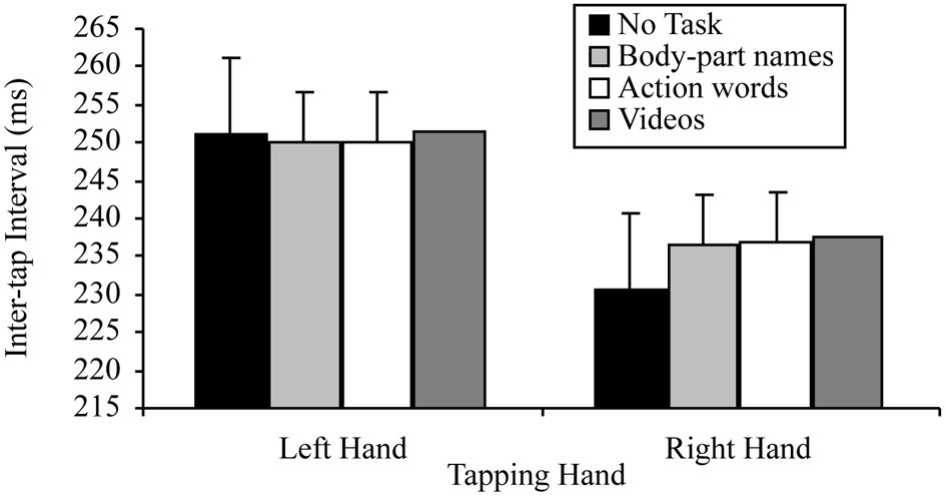
**Mean inter-tap intervals of each hand under the different concurrent task conditions (error bars represent one standard error of the mean)**.

There was a significant tapping hand × concurrent task interaction for this 2 × 4 ANOVA, *F*_(3, 72)_ = 4.53, *p* = 0.006, MSE = 33.32, part-η^2^ = 0.16. This interaction was further examined by comparing the effects of concurrent task on the right and left hand separately. The tests for the simple effects of concurrent task at the two levels of tapping hand indicated, as predicted, no effect of concurrent task on left hand tapping, *F*_(3, 72)_ = 0.63, *p* = 0.597, MSE = 21.83, part-η^2^ = 0.03, suggesting no difference between the tapping speed of the left hand regardless of whether there was no concurrent task (*M* = 250.99, SD = 41.95), reading sentences containing body-part names (*M* = 250.03, SD = 41.99), reading sentences containing action words (*M* = 250.13, SD = 41.44) or passively viewing videos (*M* = 251.59, SD = 42.80). There was, however, a significant effect of concurrent task on right hand tapping, *F*_(3, 72)_ = 4.25, *p* = 0.008, MSE = 63.59, part-η^2^ = 0.15. Simple comparisons using a Bonferroni correction to control family wise error (α = 0.05), indicated that for the right hand, as predicted, tapping was significantly slower while reading sentences containing body-part names (*M* = 236.48, SD = 44.27) or action words (*M* = 236.88, SD = 44.95) or while passively viewing videos (*M* = 237.57, SD = 47.40) than with no concurrent task (*M* = 230.46, SD = 47.99), *t*_(24, 6 comparisons)_ = −2.23, *p* = 0.035 *d* = 0.45; *t*_(24, 6 comparisons)_ = −2.27, *p* = 0.032, *d* = 0.45; *t*_(24, 6 comparisons)_ = −2.56, *p* = 0.017, *d* = 0.51, respectively. However, there was no significant difference in tapping speed between reading sentences containing body-part names (*M* = 236.48, SD = 44.27) and action words (*M* = 236.88, SD = 44.95), *t*_(24, 6 comparisons)_ = −0.27, *p* = 0.787, *d* = 0.05, or between reading either type of sentence and passively viewing videos (*M* = 237.57, SD = 47.40), *t*_(24, 6 comparisons)_ = −0.84, *p* = 0.408, *d* = 0.13; *t*_(24, 6 comparisons)_ = −0.36, *p* = 0.722, *d* = 0.07, respectively. Figure 9 summarizes these results.

#### 2 × 13 (tapping hand × semantic task) ANOVA

**Figure 10:**
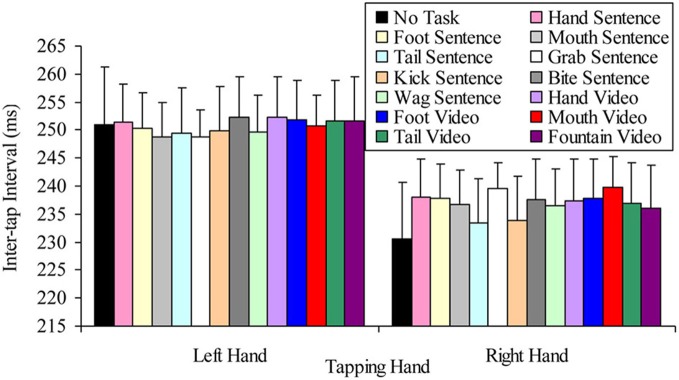
**Mean inter-tap intervals of each hand under the different concurrent semantic task conditions (error bars represent one standard error of the mean)**.

The question of whether the right hand lateralized dual task decrement would be more pronounced when participants read sentences containing hand-related words and viewed hand-related videos (compared to other semantic content), were tested by a 2 × 13 factorial repeated measures ANOVA. The variables in this analysis were: tapping hand with two levels (right vs. left); and semantic task with thirteen levels (hand sentence, grab sentence, foot sentence, kick sentence, mouth sentence, bite sentence, tail sentence, wag sentence, hand video, foot video, mouth video, tail video, fountain video). This analysis indicated a significant main effect of tapping hand *F*_(1, 24)_ = 16.23, *p* < 0.001, MSE = 1863.00, part-η^2^ = 0.40, such that left hand tapping was significantly slower (*M* = 250.37, SD = 41.71) than right hand tapping (*M* = 236.38, SD = 45.50). However, the main effect of semantic task was not significant, *F*_(12, 288)_ = 0.86, *p* = 0.588, MSE = 87.22, part-η^2^ = 0.04, suggesting no difference in tapping speed while reading sentences containing semantically different target words and viewing videos with different semantic content. The tapping hand × semantic task interaction was also not significant, *F*_(21, 288)_ = 0.79, *p* = 0.658, MSE = 63.83, part-η^2^ = 0.03. Figure 10 summarizes these results.

### Discussion

Experiment 3 was designed to test whether the null results of Experiments 1 and 2 were due to participants not associating the target words with the relevant action and body-part by supplementing the simple sentences used in Experiment 2 with videos of the actions and body-parts described by the target words. Following embodied language theories based on mirror motor neurons (Gallese and Lakoff, 2005; Rizzolatti and Craighero, 2004), it was expected that these videos would elicit visuomotor associations supporting the action-related meaning of the target words, which in turn should elicit somatotopic motor cortex activity.

As in the previous experiments, the base lateralized dual task decrement was found. A similar lateralized dual task decrement was found while participants passively viewed the videos. This might be interpreted as consistent with research on motor mirror activity in the human brain (e.g., Koski et al., 2002; Kilner et al., 2003; Lamm et al., 2007), indicating the same motor systems are activated when a given action is executed and observed. However, viewing *any* video (including that of non-biological motion) produced lateralized interference. This finding is inconsistent with the selective hand motor interference effects reported in previous mirror system investigations (e.g., Kilner et al., 2003). Alternatively, it may indicate that participants were transferring verbal labels to the actions they viewed, as the depicted body-parts/actions were referenced by the words presented in the experiment, unlike previous mirror system studies that did not include verbal conditions. Despite the base effect being found, right hand motor performance did not differ according to the semantic or lexical category of word being read. This is consistent with the findings of the previous two experiments.

## General discussion

In three experiments using the dual task paradigm we tested the hypothesis that body-part related word meanings are represented somatotopically in the motor system. Previous findings have suggested that conceptual processing of body-part related words influences subsequent movements by the specific body-parts the words refer to, and have been interpreted as indicating semantic and action representations necessarily rely on shared motor resources (e.g., Buccino et al., 2005; Lindemann et al., 2006; Sato et al., 2008; Mirabella et al., 2012). If this were the case, then comprehension of hand-related word meanings should disrupt concurrent performance of a motor task performed with the hand more so than comprehension of other body-part related words. However, this was not the case over all three experiments.

Across all three experiments, a greater decrement in right hand than left hand tapping rate was observed with concurrent word reading. This finding, referred to as the lateralized dual task decrement, was observed for single words and words embedded in sentence contexts of varying complexity. This effect was robust, occurring irrespective of whether words were read aloud or silently, thus replicating and confirming several decades of research with the dual task paradigm conducted for the purpose of investigating language lateralization (see Medland et al., 2002 for a review). The finding of a lateralized dual task decrement is evidence for the sensitivity and efficacy of the experimental manipulation as it demonstrates word reading was interfering significantly with hand motor performance (cf. Rodriguez et al., 2012). That the effect was also found when the words were read silently indicates it was not dependent on engagement of the motor articulators. Despite manipulating the body-part related meanings of the words being read, right hand tapping rates were not affected differentially by words specifically related to the hand. This suggests the lateralized dual task decrement, while typically attributed to concurrent language processing, does not reflect specific contributions from conceptual processing of words relating to body-parts.

Several alternate explanations were explored for the findings that concurrent reading of hand related words did not affect right hand tapping rates more than words relating to other body-parts. These included the low complexity of the verbal stimuli resulting in a less pronounced overall lateralized dual task effect, semantic satiation, and a weakened association between the target word and the action/body-part it described. However, no support was found for these possible explanations, as neither embedding the words in sentence contexts nor presenting them in conjunction with videos depicting the actions/body-parts the words referred to elicited individual word effects consistent with body-part meanings being represented somatotopically on the motor cortices. Another possible explanation for the results might be that multiple representations of action meanings related to a specific body-part need to be maintained in working memory in order for semantic activity to achieve a threshold level of activity capable of influencing motor performance (e.g., Shebani and Pulvermüller, 2013; cf. Rodriguez et al., 2012). However, if this were the case, then one would not expect to observe effects with single words in the go/no-go paradigm (e.g., Lindemann et al., 2006; Sato et al., 2008; Mirabella et al., 2012). In addition, if maintaining and manipulating multiple representations of the same type in working memory is required to demonstrate a semantic somatotopy, then it is arguably an example of context-dependent activation and certainly not an automatic process. Finally, we conducted *post-hoc* power analyses on the data of all omnibus tests in Experiments 1–3 (D'Amico et al., 2001). For the 2 × 3 ANOVAs in Experiments 1–2 and the 2 × 4 ANOVA in Experiment 3, the interactions of tapping hand and reading condition showed levels of power above the recommended 0.80 level (see Cohen, 1992). Therefore, the non-significant effects possessed sufficient power to be retained as null results, and a Type II error is unlikely to have occurred.

In conclusion, these findings from the dual-task paradigm all support the view that motor activity observed in association with action word comprehension is context/task-dependent or epiphenomenal, reflecting the flow of activation between essentially separate conceptual and motor systems (e.g., Mahon and Caramazza, 2008; Postle et al., 2008). While motor simulation may play a functional role in performance of some tasks such as the go/no-go paradigm that require post-lexical semantic matching/meaning integration, the findings with the dual task paradigm indicate this role is neither a necessary nor automatic one. More generally, the findings demonstrate the continued utility of the dual task paradigm for investigating interactions between language and motor processes.

### Conflict of interest statement

The authors declare that the research was conducted in the absence of any commercial or financial relationships that could be construed as a potential conflict of interest.
